# Subtype-Specific Alterations in Copper Trafficking Associated with KRAS Mutations in Isogenic Colorectal Cancer Cell Lines

**DOI:** 10.1007/s12011-026-05171-w

**Published:** 2026-06-02

**Authors:** Elina Üveges, Anikó Gaál, Gergely Szakács, Éva Bakos, Christina Streli, Peter Kregsamer, Marcell Baranyi, Balázs Hegedűs, Norbert Szoboszlai

**Affiliations:** 1 Integrative Health and Environmental Analysis Research Laboratory, Institute of Chemistry, ELTE Eötvös Loránd University, Budapest, Hungary; 2 Semmelweis University Doctoral School, Division of Pharmaceutical Sciences and Health Technologies, Semmelweis University, Budapest, Hungary; 3 Biological Nanochemistry Research Group, Institute of Materials and Environmental Chemistry, Research Centre for Natural Sciences, Magyar tudósok körútja 2, Budapest, 1117 Hungary; 4 Institute of Molecular Life Sciences, HUN-REN Research Centre for Natural Sciences, Budapest, Hungary; 5 Center for Cancer Research, Medical University Vienna, Vienna, Austria; 6 Institute of Atomic and Subatomic Physics, Vienna University of Technology, Vienna, Austria; 7 X-ray center TU Wien, Vienna, Austria; 8 Department of Pathology, Forensic and Insurance Medicine, Semmelweis University, Budapest, Hungary; 9 Department of Thoracic Surgery, University Medicine Essen – Ruhrlandklinik, Essen, Germany

**Keywords:** Copper, Colorectal cancer, Chelating agent, KRAS mutation, Copper transporter

## Abstract

Copper bioavailability has been identified as a potential target for KRAS-mutant colorectal cancer, a disease for which treatment options remain limited. Therefore, we investigated copper homeostasis in isogeneic colorectal cancer cell models carrying distinct KRAS codon 12 mutations (G12C, G12D and G12V). Our results show that copper homeostasis differs among KRAS-mutant cells in a mutation-specific manner, with the greatest change observed in G12V, followed by G12C and then G12D. Specifically, G12V-mutant cells show reduced copper levels, increased copper uptake, reduced intracellular copper levels after prolonged exposure to exogenous copper, reduced sensitivity to copper-induced toxicity, protection against intracellular copper chelators (neocuproine, DpC, Dp44mT), and increased expression of the copper transporter ATP7A. In contrast, G12D mutant cells exhibit minimal differences in copper metabolism compared to controls, while G12C display an intermediate phenotype between the other two mutations. Notably, the copper chelator ammonium tetrathiomolybdate inhibited cell growth in all KRAS-mutant cells. Our results suggest that KRAS-mutant colorectal cancer cells are sensitive to perturbations in copper homeostasis in a mutation subtype-specific manner.

**Supplementary Information:**

The online version contains supplementary material available at 10.1007/s12011-026-05171-w.

## Introduction

KRAS mutations are among the most frequent oncogenic mutations in human tumors [1]. They occur in more than 80% of pancreatic adenocarcinomas [2], in approximately 30% of colorectal cancers (CRC) where KRAS is the most commonly mutated RAS gene [3], and up to 30% of lung adenocarcinomas [4]. KRAS mutations are also detected in endometrial and ovarian cancers, with an incidence exceeding 10% [2]. Despite the established significance of these mutations, effective therapy of KRAS-mutant tumors remains a major clinical challenge. The most common subtypes of KRAS mutations are G12D, G12V and G12C. Over recent decades, no effective targeted therapies have been developed, with the exception of two drugs targeting the G12C variant: sotorasib, approved for lung cancer, and adagrasib, approved for lung cancer and colorectal cancer patients harbouring KRAS G12C mutations [5–7]. The KRAS gene is the human homologue of the Kirsten rat sarcoma viral oncogene, which is a member of the RAS family. Mutation of KRAS results in aberrant activation of multiple signaling pathways associated with cell proliferation, metastasis, altered metabolism and microenvironment, as well as enhanced cell survival [8, 9].

Earlier studies have shown altered copper trafficking in KRAS mutated CRC cells and MEF (mouse embryonic fibroblast) cells. Specifically, KRAS G12V cells exhibit elevated levels of bioavailable copper, as well as increased expression of the copper efflux transporter ATP7A (Menkes’ protein) [9]. In contrast, in another study, decreased labile copper pool levels were observed in KRAS G12D cells with a ratiometric fluorescence resonance energy transfer (FRET) probe [10]. KRAS G12D cells were also shown to have elevated intracellular Cu(II) levels and decreased Cu(I) levels suggesting a shift toward a more oxidizing intracellular environment associated with oncogenic transformation [11].

Two key transporters, ATP7A and ATP7B (Wilson disease protein) are responsible for maintaining intracellular copper homeostasis. These transporters are located in the trans-Golgi network and translocate to the cell membrane to mediate copper efflux whenever excess copper is present in the cell. ATP7A appears to have an important role in KRAS mutated cell lines [9, 12]. Copper uptake primarily occurs via the CTR1 transporter, though it was shown to be dispensable in KRAS mutated cells. Divalent metal transporter 1 (DMT1), primarily known as an iron transporter, can also import copper under certain circumstances; however it has been shown to play only a compensatory role in copper uptake in the absence of CTR1 [13]. In addition to transporter-mediated uptake, nutrients may also enter cells via macropinocytosis, a process whose relevance is currently being intensively investigated in KRAS-mutant cells [14]. Identifying metabolic alterations and differences in transport mechanisms between wild-type and KRAS-mutant cells is key to the effective targeting of cancer cells. A deeper understanding of how copper metabolic processes change in tumor cells may facilitate the development of new treatments for cancer therapy. Given the limited number of comparative studies addressing the interplay between KRAS mutations and copper metabolism, our aim was to establish a comprehensive experimental model to evaluate and compare the effects of the three most frequent KRAS mutations on copper metabolism.

## Materials and Methods

### Chemicals and Cell Lines

Throughout the experiments, deionized Milli-Q (Millipore, Molsheim, France) water with a relative conductivity of 18.2 MΩ cm was used. All the chemicals were of analytical grade, unless stated otherwise. The following chemicals were purchased from Sigma Aldrich Ltd. (Budapest, Hungary): Copper(II) chloride, magnesium chloride, sodium chloride, 2-[4-(2-hydroxyethyl)piperazin-1-yl]ethanesulfonic acid (HEPES), ascorbic acid, and the following copper chelating agents: di-2-pyridylketone-4,4,-dimethyl-3-thiosemicarbazone (Dp44mT), neocuproine, di-2-piridylketone 4-cycloheyl-4-methyl-3-thiosemicarbazone (DpC) and ammonium tetrathiomolybdate (TTM). Dextran- fluorescein isothiocyanate conjugate (dextran-FITC) needed for the fluorescent activated cell sorting (FACS) measurements were purchased from Merck (Darmstadt, Germany). Concentrated nitric acid (65% w/w) and H_2_O_2_ (30% w/w) of Suprapur quality needed for the sample preparation of cell lines for Total-Reflection X-ray Fluorescence (TXRF) measurement were supplied by Merck (Darmstadt, Germany).

The following human cancer cell lines were used throughout the experiments: SW48, SW48 G12C, SW48 G12D, SW48 G12V colon adenocarcinomas. Isogenic SW48 cells were obtained from Horizon Discovery and were generated by a heterozygous knock-in of a KRAS activating mutation with recombinant Adeno-Associated Virus-based gene editing [15]. Presence of the mutations were validated at Semmelweis University, Department of Pathology, Forensic and Insurance Medicine by sequencing.

### Presto Blue™ Cell Viability Assay

Cells were grown at 37 °C in a humidified atmosphere of 5% CO_2_ in Dulbecco’s Modified Eagle Medium (DMEM) containing 4,500 mg/L glucose (Sigma Aldrich) supplemented with 10% v/v foetal bovine serum (FBS, Sigma Aldrich). Twenty-four hours before the treatment, cells were plated into a 96-well flat bottom culture plate (with initial 5000 cell/100 µL DMEM medium/well). After 24 h incubation at 37 °C, 100 µL of the test compounds were added to the cells in serial two-fold dilutions. Cells were then incubated with the test compounds for 72 h. After washing the cells with PBS (phosphate buffered saline), cell viability was assessed using the Presto Blue™ assay (Life Technologies) following the manufacturer’s instructions.

### Cell Growth and Determination of Doubling Time

Cells were grown at 37 °C in a humidified atmosphere of 5% CO_2_ in Dulbecco’s Modified Eagle Medium (DMEM) containing 4,500 mg/L glucose (Sigma Aldrich) supplemented with 10% v/v foetal bovine serum (FBS, Sigma Aldrich). For the doubling time experiments, cells were cultured in 12-well Thermo Fisher Scientific culture plates (5 × 10^5^ cells/well). After 24 h, 48 h, 72 h, 96 h, 120 h, 144 h, and 168 h, cells were harvested with a trypsin–EDTA solution (5.0 g/L porcine trypsin and 2.0 g/L EDTA·4 Na in 0.9% v/v sodium chloride solution purchased from Sigma Aldrich). Trypsinization was stopped by dilution with completed DMEM (+ 10% FBS) and cell numbers/mL were determined using trypan blue, with a Beckman Coulter Vi-CELL XR Cell Viability Analyzer. Cell doubling time (*T*_d_) was calculated assuming exponential growth using the following equation:

*T*_d_ = ln(2)/*k*.

where *k* is the exponential growth rate constant obtained by the slope of a nonlinear regression of the exponential growth phase. Fits and calculations were performed in GraphPad Prism 8.0.1.

### Sample Preparation for TXRF Measurements

Intracellular content of Cu was determined by a total-reflection X-ray fluorescence (TXRF) method as reported elsewhere [16, 17]. Samples were cultured in 6-well Thermo Fisher Scientific culture plates (5 × 10^5^ cells/well). Cells were incubated overnight and subjected to copper(II) chloride treatment in completed DMEM. The treatment concentrations were 0 or 2 µM or 5 µM copper(II) chloride for the 72 h experiments. For the 2 h experiments, samples were cultured in 6-well Thermo Fisher Scientific culture plates (1.5 × 10^6^ cells/well), incubated overnight, and subjected to 0, 5, 10, 20 and 50 µM copper(II) chloride treatment for 2 h in uptake buffer. The uptake buffer was freshly prepared for each experiment by dissolving NaCl (150 mM) MgCl_2_ (2.5 mM) and HEPES sodium salt (25 mM) in water, adjusting the pH to 7.4, and filtering through a 0.22 μm membrane filter. For certain samples, ascorbic acid (10 µM) was added to the uptake buffer right before copper treatment. After a 72 h–2 h long incubation, cells were harvested with a trypsin–EDTA solution. Trypsinization was stopped by dilution with completed DMEM (+ 10% FBS) and cells were washed twice with 1 mL of PBS (Sigma Aldrich). Following centrifugation, the supernatant was removed and the cell pellets were digested by adding 20 µL of 30% H_2_O_2_, 80 µL of 65% HNO_3_ and 15 µL of 10 µg/mL Ga, followed by incubation for 24 h at room temperature.

### Total Reflection X-Ray Fluorescence Spectrometry (TXRF)

The TXRF analyses were performed by using a TXRF 8030 C spectrometer (Atomika Instruments GmbH, Oberschleissheim, Germany), equipped with a 2.5 kW X-ray tube made of a Mo/W alloy anode and a double-W/C multilayer monochromator, adjusted to obtain an excitation energy of 17.4 keV (Mo K_α_). In this equipment, the characteristic radiation emitted by the elements present in the sample is detected by a Si(Li) detector with an active area of 80 mm^2^ and a resolution of 150 eV at 5.9 keV. The measurements were performed working at 50 kV and the current was adjusted automatically as a trade-off between the detector dead time and total analysis time. The acquisition time was 500s. Gallium was used as the internal standard. Two microliters from the samples were pipetted onto quartz reflectors and dried at 80 °C on a ceramic coated heater (Cole-Parmer, USA) prior to analysis. The K_α_ line used for determination of Cu was 8.047 keV.

### ATP7A, CTR1 and DMT1 Protein Expression Measurement

Cells were grown at 37 °C in a humidified atmosphere of 5% CO_2_ in Dulbecco’s Modified Eagle Medium (DMEM) containing 4,500 mg/L glucose (Sigma Aldrich) supplemented with 10% v/v fetal bovine serum (FBS, Sigma Aldrich). Samples were cultured in 6-well Orange Scientific Tissue culture plates 5 × 10^5^ cells/well). Cells were incubated overnight and subjected to copper(II) chloride treatment. The treatment concentrations were 0, 2 and 5 µM copper(II) chloride respectively. After a 72 h long incubation, cells were harvested with a trypsin–EDTA solution (5.0 g/L porcine trypsin and 2.0 g/L EDTA·4 Na in 0.9% v/v sodium chloride solution purchased from Sigma Aldrich). Trypsinization was stopped by dilution with completed DMEM (+ 10% FBS) and cells were washed twice with 1 mL of PBS (Sigma Aldrich). After centrifugation, the supernatant was removed and 200 µl of cell lysis buffer was added to the samples. Protein concentration of the samples was determined by the Lowry method [18], ATP7A (clone S60-4) and CTR1 (clone 4C10C5) monoclonal antibodies, and DMT1 polyclonal antibody (20507-1-AP) were purchased from Thermo Fisher Scientific. ATP7A, CTR1 and DMT1 protein expression was measured by immunoblot [19]. Samples were loaded into an SDS-PAGE gel for protein separation and transferred to a PVDF membrane, antibodies were diluted (1:1000) in a blocking solution and detected using ChemiDoc™ imaging system with chemiluminescent detection. The band densities were quantified by densitometry using Image Lab software (Bio-Rad Laboratories). To quantify the western blotting data, the intensities of ATP7A in each gel were normalized to their respective actin intensities, then the normalized intensities were compared with the intensity of untreated KRAS wild-type cells and expressed as relative values to it.

### Macropinocytosis Measurement with Fluorescence-Activated Cell Sorting

Cells were grown at 37 °C in a humidified atmosphere of 5% CO2 in Dulbecco’s Modified Eagle Medium (DMEM) containing 4,500 mg/L glucose (Sigma Aldrich) supplemented with 10% v/v fetal bovine serum (FBS, Sigma Aldrich). Samples were cultured in 24-well Thermo Fisher Scientific culture plates (3 × 10^5^ cells/well). Cells were incubated overnight and treated either with dextran-FITC (Mw: 70 kDa, dilution: 1 mg/ml) for 1 h in uptake buffer (150 mM NaCl, 2.5 mM MgCl_2_, 25 mM HEPES, pH = 7.4), or with vehicle. Fluorescence intensity was measured with a BD FACSCalibur flow cytometer.

### Bioinformatic Analysis

For transcript level analysis, we investigated two databases. We analyzed the ATP7A mRNA levels in various KRAS-mutant colorectal cancer cases from the TCGA colorectal cancer cohort downloaded through cBIOPORTAL. Second, we compared the impact of mRNA levels on recurrence-free survival in a large combined cohort of colorectal cancer cases in the KMPlot database. The optimal cut-off was determined as the best performing cut-off among all possible values between the lower and upper quartiles.

## Results

### Proliferation of Isogenic KRAS-Mutant SW48 Cells

Copper trafficking was analyzed in a SW48 colorectal cell line panel including SW48 KRAS wild-type, SW48 G12C, SW48 G12D and SW48 G12V KRAS-mutant cell lines [20]. First, we compared cell morphology in sub-confluent cells and observed no obvious morphological differences among the four cell lines. Next, we assessed cell proliferation by determining doubling times of each cell line using an automated cell counter. The doubling time was 33 h, 29.5 h, 45.3 h, and 34.5 h for SW48 WT, SW48 G12C, SW48 G12D, and SW48 G12V cells, respectively. Notably, the G12D mutant — representing the most frequent KRAS mutation in human tumors — exhibited significantly slower growth, suggesting that this mutation may alter cellular pathways influencing proliferation.

### Baseline Copper Levels of SW48 KRAS-Mutant Cell Lines

**Fig. 1:**
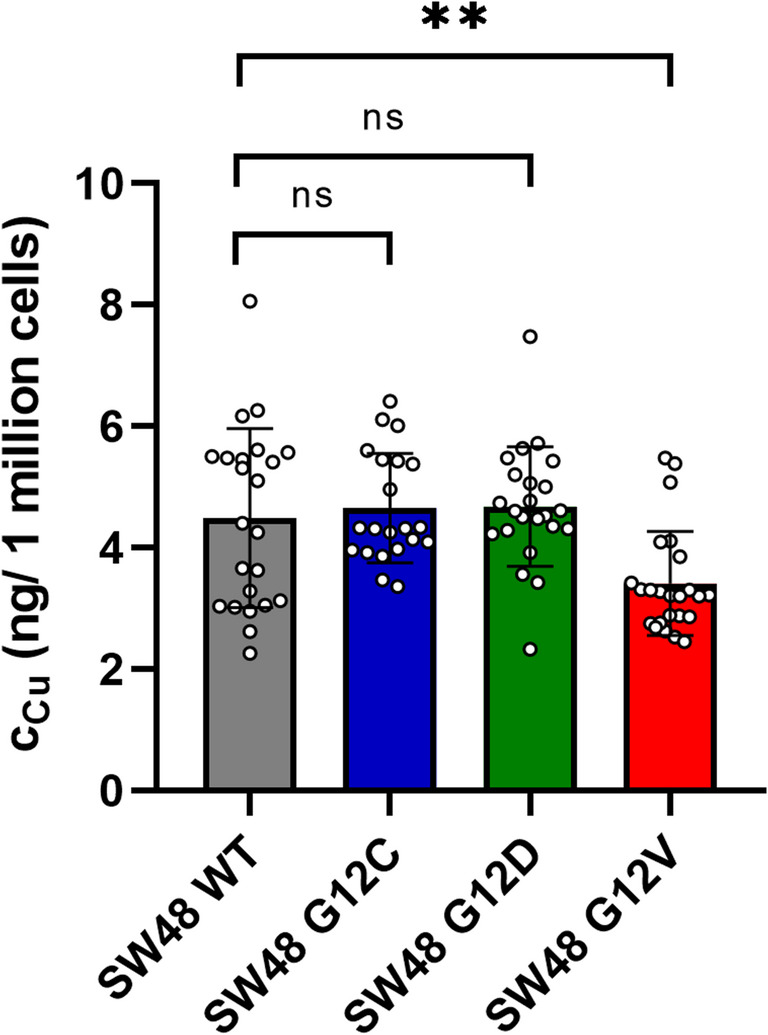
Baseline copper concentrations of different SW48 cell lines. Copper levels are significantly lower in SW48 G12V cells, compared to the other three subtypes. The results are based on 24 independent measurements. Significance levels were determined using one-way ANOVA with Tukey’s multiple comparisons test: *p < 0.05, **p < 0.01 when compared to the KRAS wild-type cells

Copper uptake and related intracellular processes are clinically relevant, as altered copper homeostasis has been linked to cancer development in several recent studies [21–26]. In our study, we measured baseline copper concentrations in SW48 cells with the TXRF method [16]. Average copper content of the G12V cell line was 3.4 (± 0.85) ng per one million cells, and it ranged from 4.4 (± 0.7) ng to 4.7 (± 1.0) ng per one million cells in wild-type, G12C and G12D cell lines. Due to the large standard deviation in the copper content data measured in the cells, it was necessary to analyze a large number of sample replicates (*n* = 24). Based on our results, the copper content of G12C and G12D cells did not differ significantly, whereas KRAS G12V cells exhibited significantly lower levels compared to wild-type cells (Fig. 1).

### Copper Uptake in SW48 Cells under Normal and Nutrient-Deprived Conditions

During copper uptake measurements, cells are typically exposed to varying concentrations of copper (especially when chelator-mediated copper uptake is assessed) for four hours in DMEM to monitor metal ion accumulation [27]. While this approach is effective for many cell lines [28], no significant copper uptake was observed in SW48 cells, even at higher copper concentrations, indicating that copper was not bioavailable under these conditions. Furthermore, this pattern was not altered by KRAS mutations, as none of the mutations increased the copper uptake.

**Fig. 2:**
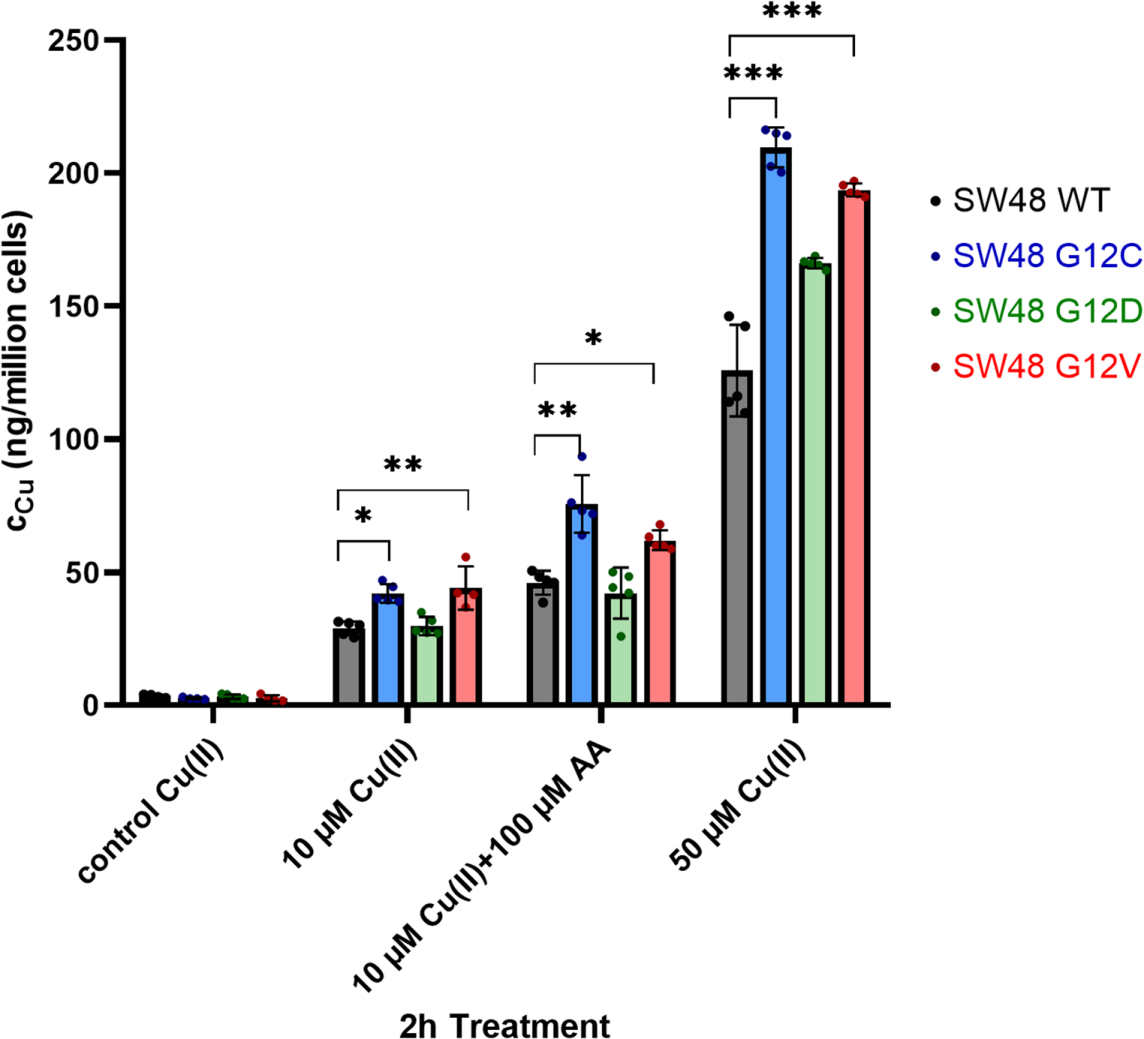
Copper uptake in KRAS wild-type and mutant cells following short-term CuCl_2_ treatment. Intracellular copper levels were measured in KRAS wild-type and mutant SW48 cells (G12C, G12D, and G12V) after a 2 h incubation in uptake buffer with low concentrations of CuCl₂, in the presence or absence of ascorbic acid, using TXRF analysis. The treatments included 10 µM CuCl₂ alone, 10 µM CuCl₂ supplemented with 100 µM ascorbic acid, and 50 µM CuCl₂ alone. Data represents three independent experiments, each performed with two technical replicates. Statistical significance was assessed using one-way ANOVA with Tukey’s multiple comparisons test: *p < 0.05, **p < 0.01, ***p < 0.001 when compared to the KRAS wild-type cells

Certain components of DMEM, particularly amino acids such as histidine and tyrosine, can form stable complexes with Cu ions, thereby limiting their cellular accessibility. Therefore, we repeated the experiments to eliminate the growth medium and simulate nutrient-deprived conditions using a special uptake buffer commonly employed in the literature (150 mM NaCl, 2.5 mM MgCl_2_, 25 mM HEPES, pH = 7.4) [29]. These short-term uptake experiments (Fig. 2) showed that KRAS G12C and G12V mutant cells have more intensive copper uptake compared to wild-type cells under nutrient-deprived conditions, whereas the difference between KRAS G12D and wild-type cells was not significant. It is not yet clear which transport mechanism is responsible for copper uptake, and in what form copper enters these colorectal cells. Several studies have proposed that macropinocytosis, an endocytic pathway upregulated in KRAS-mutant cancer cells, serves as a major route for copper entry [14]. In addition, certain copper transporters (e.g. CTR1) import the reduced form of copper via active transport [15]. To assess whether copper reduction influences uptake, we co-administered ascorbic acid (AA), a reducing agent with copper(II) chloride in the uptake buffer (CuCl_2_: AA, 1:10) for 2 h. Copper uptake increased in all cell lines, however, the elevation in intracellular copper concentrations was more pronounced in SW48 G12C and G12V cells compared to the wild-type or G12D cells following ascorbic acid treatment (Fig. 2). These results indicate that uptake is enhanced when copper is in a reduced form, suggesting that a transport mechanism may be involved in copper uptake under nutrient-deprived conditions.

### Response of SW48 Cells to Prolonged (72 h) Low-Concentration Copper Exposure

**Fig. 3:**
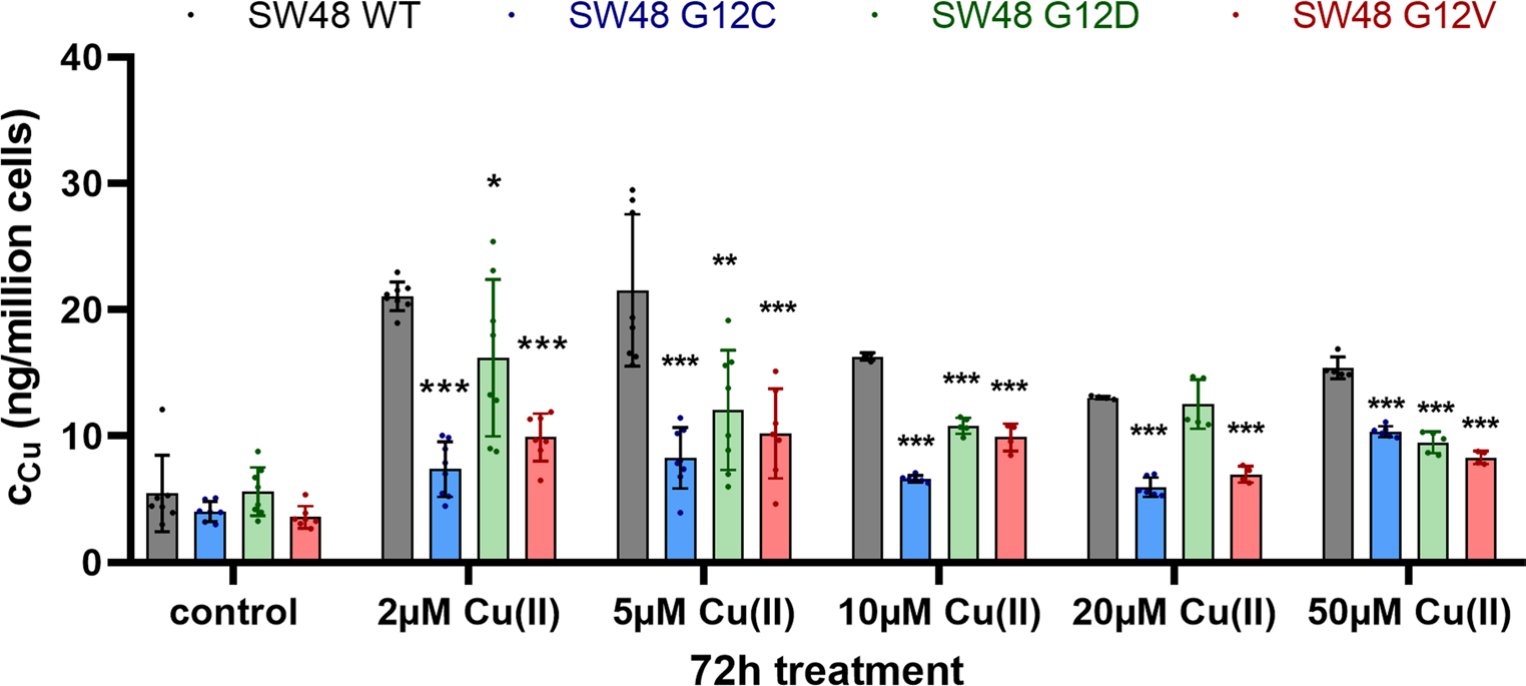
Copper concentrations of KRAS wild-type cells, compared to KRAS-mutant cells (G12C, G12D, and G12V) after 72 h low concentration CuCl_2_ treatment, measured with the TXRF method. The results are based on 4 independent measurements, with 2 technical replicates in each measurement. Significance levels were determined using one-way ANOVA with Tukey’s multiple comparisons test: *p < 0.05, **p < 0.01, ***p < 0.001 when compared to the KRAS wild-type cells

**Fig. 4:**
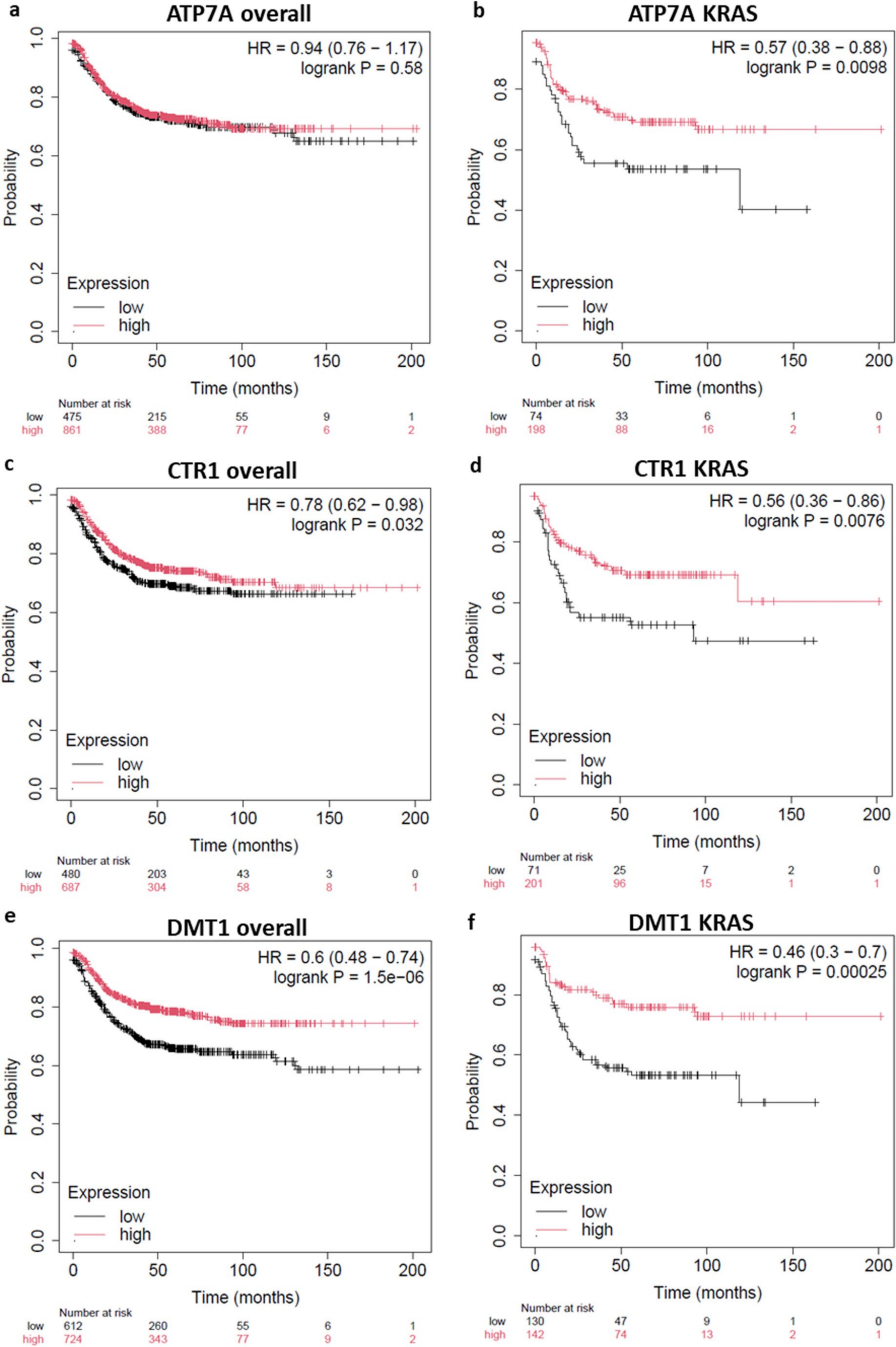
Association of ATP7A (**a-b**), CTR1 (**c-d**) and DMT1 (**e-f**) transcript levels with recurrence-free survival (RFS) in colorectal cancer patients (*N* = 1336), and in the KRAS-mutant subcohort (*N* = 272). Low ATP7A transcript levels are associated with lower RFS only in KRAS-mutant patients. Low CTR1 and DMT1 transcript levels are associated with lower RFS in all patients, however, the difference is more pronounced in the KRAS-mutant subcohort

Several studies have reported that copper metabolism is altered in KRAS-mutant cells, and various copper-related proteins and intracellular processes may contribute to this alteration. We therefore tested how these changes influence the response of KRAS-mutant cells to prolonged copper exposure. Our results showed that, after 72 h of low-concentration copper(II) chloride treatment, intracellular copper levels were significantly higher in SW48 KRAS wild-type cells compared to SW48 KRAS-mutant cells (Fig. 3). These findings suggest the presence of a mechanism in KRAS-mutant cells that either limits copper uptake or enhances copper efflux. According to the literature, the copper transporter ATP7A is upregulated in colorectal KRAS-mutant cells; however, this observation is based on a single study comparing KRAS G12V and wild-type cells [9, 12]. In order to investigate the potential clinical relevance of copper transporters including ATP7A in KRAS-mutant colorectal cancer, we compared the association of ATP7A, CTR1, DMT1 transcript levels in colorectal cancer tissue with recurrence-free survival (RFS) in a large database of colorectal cancer patients and in the KRAS mutation carrying subcohort [30]. While ATP7A levels had no prognostic impact in the overall cohort, low ATP7A expression in KRAS-mutant patients was significantly associated with shorter RFS (Fig. 4). As our results revealed distinct patterns of copper metabolism among the three KRAS-mutant cell lines, we next examined ATP7A expression in all four cell lines.

### Expression of ATP7A, CTR1 and DMT1 in KRAS-Mutant and Wild-Type Cells

**Fig. 5:**
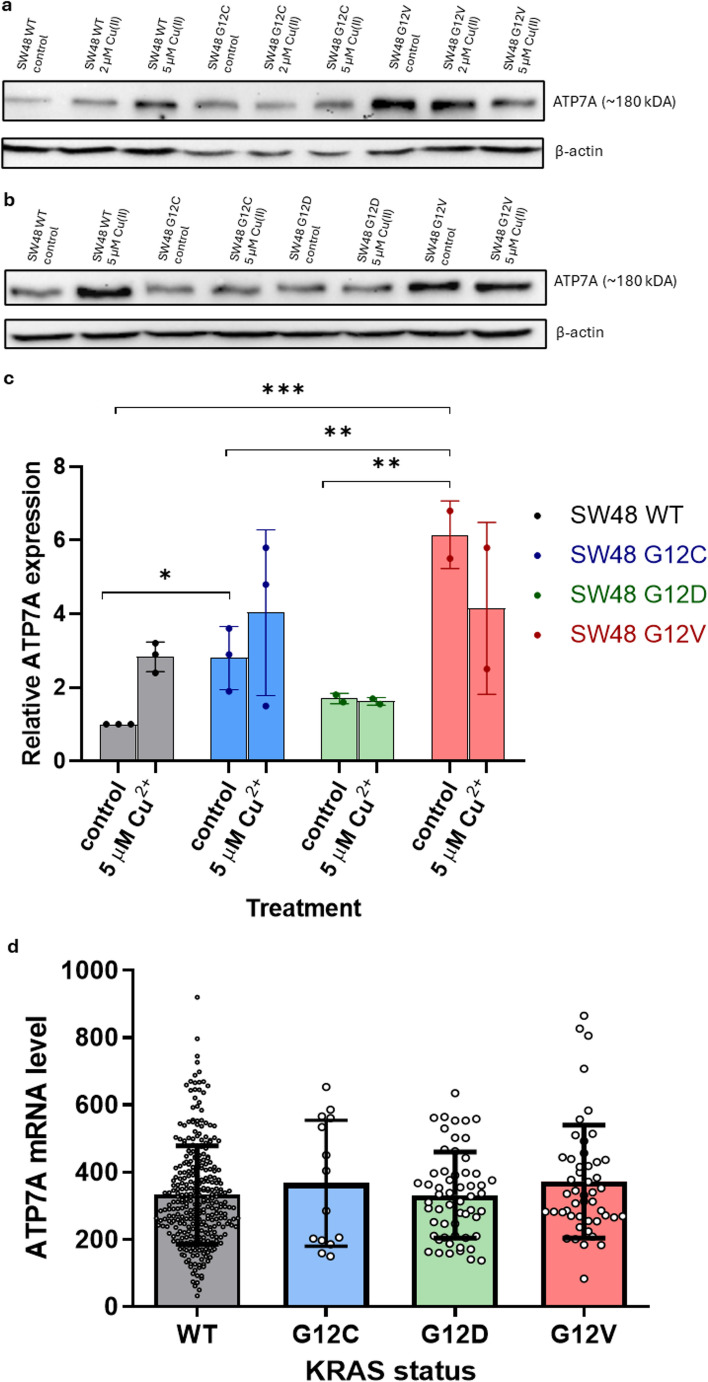
ATP7A protein and mRNA expression in SW48 cells carrying different KRAS mutations following copper treatment. **a-b** Representative Western blots showing ATP7A protein expression in KRAS wild-type and mutant SW48 cells after 72 h copper treatment. In KRAS wild-type cells, ATP7A expression was low under basal conditions and increased following copper exposure. KRAS G12C cells exhibited moderately elevated ATP7A expression in the absence of treatment, whereas KRAS G12D cells showed generally lower ATP7A levels. In contrast, KRAS G12V cells displayed high ATP7A expression both with and without copper treatment. **c** Densitometric quantification of ATP7A protein levels normalized to β-actin and expressed relative to untreated KRAS wild-type cells (set to 1). Data represent three independent experiments and are shown as mean ± SD, significance levels were determined using one-way ANOVA with Tukey’s multiple comparisons test: (*p < 0.05, **p < 0.01, ***p < 0.001). **d** ATP7A mRNA expression across KRAS mutation subtypes in colorectal cancer samples from The Cancer Genome Atlas (TCGA) database. ATP7A transcript levels were modestly elevated in KRAS G12C and G12V tumors. A total of 433 samples were analyzed, including KRAS wild-type (*n* = 314), KRAS G12C (*n* = 14), KRAS G12D (*n* = 57), and KRAS G12V (*n* = 48)

ATP7A plays a major role in copper metabolism of colorectal cells. Its expression is typically upregulated under high copper conditions, protecting cells from copper-induced cytotoxicity. Previous studies have reported elevated ATP7A levels in KRAS G12C-mutant lung adenocarcinoma cells [31] as well as in KRAS G12V intestinal epithelial cells [9], whereas KRAS G12D mouse embryonic fibroblasts displayed lower ATP7A transcript levels compared to control cells [10]. Because these observations were made in different cell types, our goal was to assess ATP7A transcript levels across all four SW48 cell lines, allowing a direct comparison of the effects of distinct KRAS point mutations within a consistent cellular context. Our results show that wild-type cells exhibit low ATP7A expression under basal conditions, which increases following copper treatment. In contrast, KRAS G12C and KRAS G12V cells displayed elevated ATP7A expression both before and after copper exposure, with the highest levels observed in KRAS G12V cells (Fig. 5a). Interestingly, the analysis of a colorectal adenocarcinoma patient cohort in The Cancer Genome Atlas (TCGA) revealed a similar trend, with increased ATP7A expression in KRAS G12V- and KRAS G12C-mutant tumors compared to KRAS WT or KRAS G12D cases (Fig. 5d) [32]. Under basal conditions, KRAS G12V cells exhibited significantly higher ATP7A expression than KRAS G12C cells, although ATP7A levels in KRAS G12C cells were also significantly elevated relative to wild-type controls. In contrast, KRAS G12D cells showed ATP7A expression comparable to that of wild-type cells (Fig. 5b). These findings are consistent with previously published data and are here confirmed in isogeneic SW48 cell model carrying distinct KRAS mutations.

**Fig. 6:**
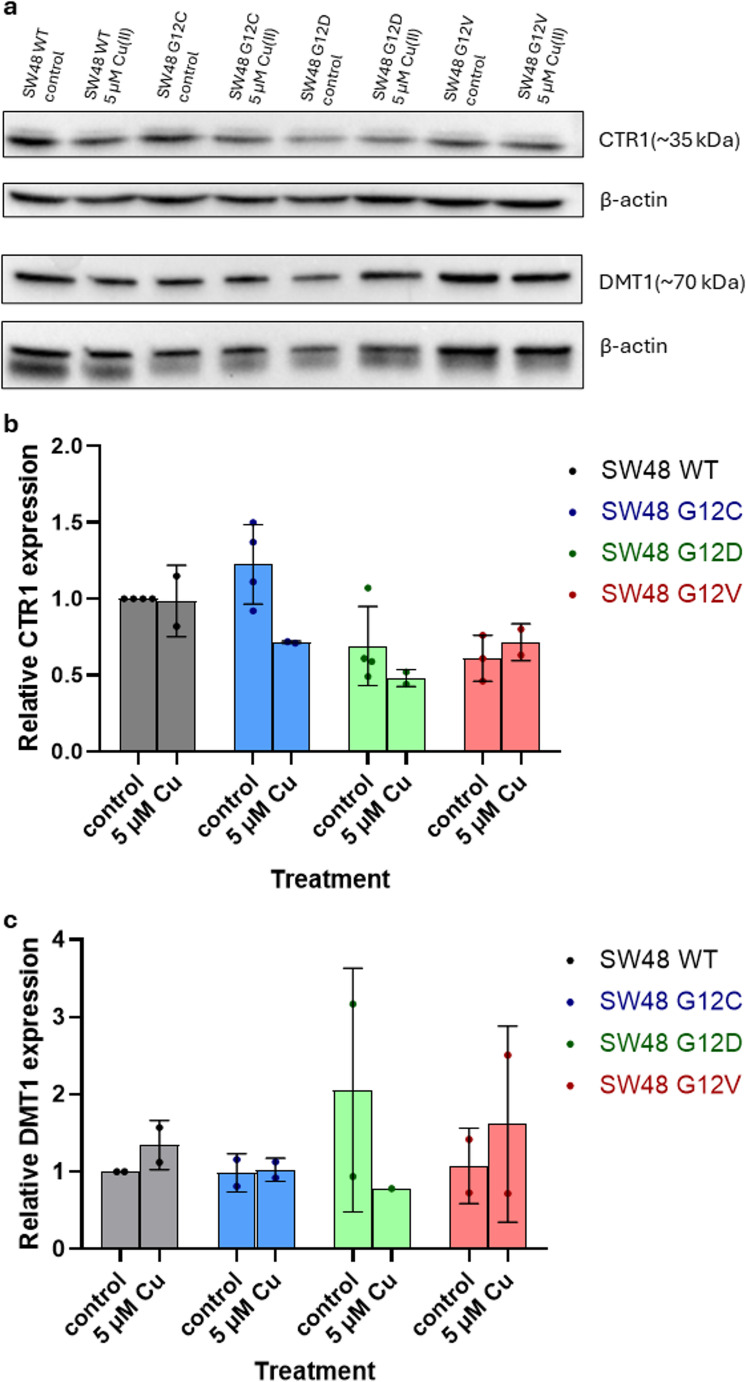
Protein expression of the copper importers CTR1 and DMT1 in SW48 cells carrying different KRAS mutations following 72 h copper treatment.** a** Western blot analysis of the copper importers CTR1 and DMT1 in KRAS wild-type and mutant SW48 cells without treatment and after 72 h copper treatment. CTR1 and DMT1 proteins were detected in all four cell lines, with no notable increase in expression in KRAS-mutant cells compared to wild-type controls. **b-c** Densitometric analysis of the expression of CTR1 and DMT1 proteins. For CTR1, four replicates were used to assess protein expression under basal conditions, while two replicates were analyzed following 72-hour copper(II) treatment. For DMT1 expression analysis, two replicates were used and analyzed. There are no significant differences between the cell lines in the expression of either protein

Previous studies have shown that CTR1 function is essential for tumor survival in KRAS G12D lung cancer cells [33, 34]. To determine whether CTR1 expression differed among the four SW48 sublines, we assessed CTR1 protein levels before and after copper exposure. CTR1 was detected in all four cell lines under both conditions, with no increase in expression observed in the KRAS-mutant cells compared to wild-type cells (Fig. 6a). However, differences between wild-type and mutant cells may still exist at the level of CTR1 subcellular localization or post-translational regulation.

Our experiments further demonstrated that short-term copper uptake is more pronounced in SW48 G12C and G12V cells. To assess whether another active transporter might contribute to this effect, we examined the expression of DMT1. Similar to CTR1, no elevated DMT1 expression was detected in the KRAS-mutant SW48 cells (Fig. 6a and c).

### Comparison of Macropinocytosis Intensity in SW48 Wild-Type and Mutant Cells

**Fig. 7:**
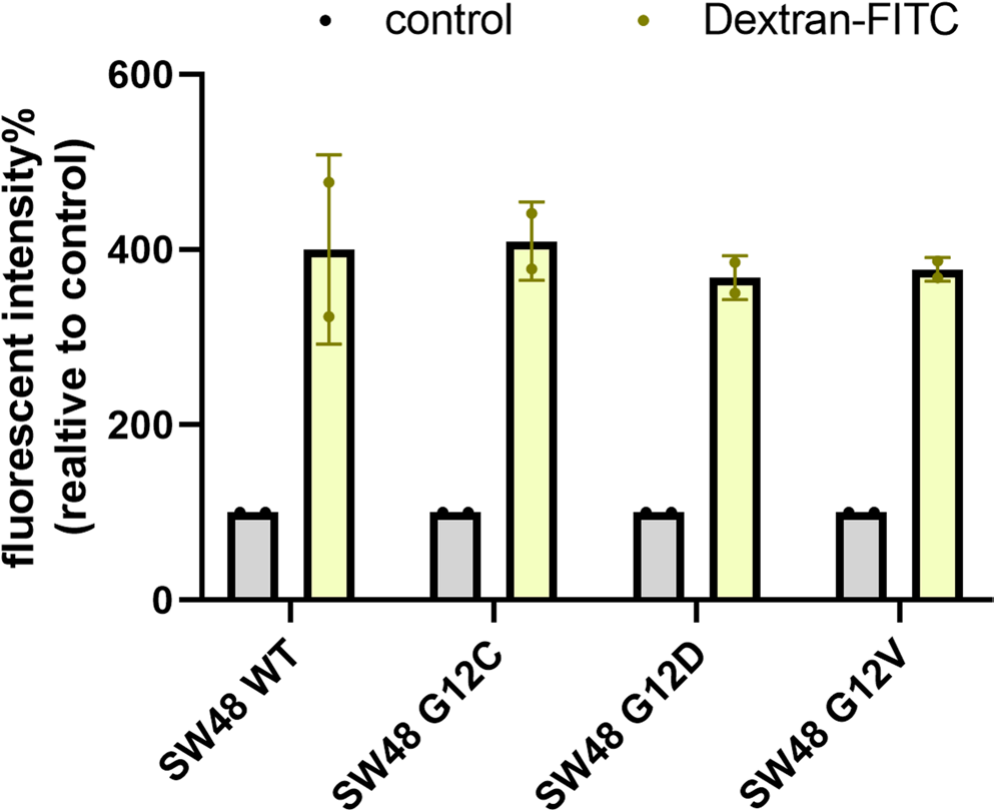
Relative fluorescent intensity of SW48 wild-type and KRAS-mutant cells following a 1 h incubation with 1 mg/mL FITC–dextran in uptake buffer, measured by fluorescence-activated cell sorting (FACS). Based on one-way ANOVA analysis, no significant differences in macropinocytic uptake were observed between the cell lines. Shown results represent two individual experiments

We evaluated the intensity of macropinocytosis using TMR-dextran uptake measured by flow cytometry [35]. Repeated experiments revealed no detectable differences between KRAS-mutant and wild-type SW48 cells regardless of changes in the duration of treatment or starvation conditions. The results presented in Fig. 7 reflect fluorescence intensity measured following a 1-hour treatment in uptake buffer, corresponding to the conditions used in the copper uptake experiments. These results suggest that enhanced macropinocytosis is observed in wild-type KRAS cells and is not further increased in mutant cells, suggesting that it does not explain the elevated copper uptake observed in SW48 G12C and G12V cells.

### Effects of Nutrient Deprivation and Copper Exposure on the Survival of SW48 Wild-Type and KRAS-Mutant Cells

**Fig. 8:**
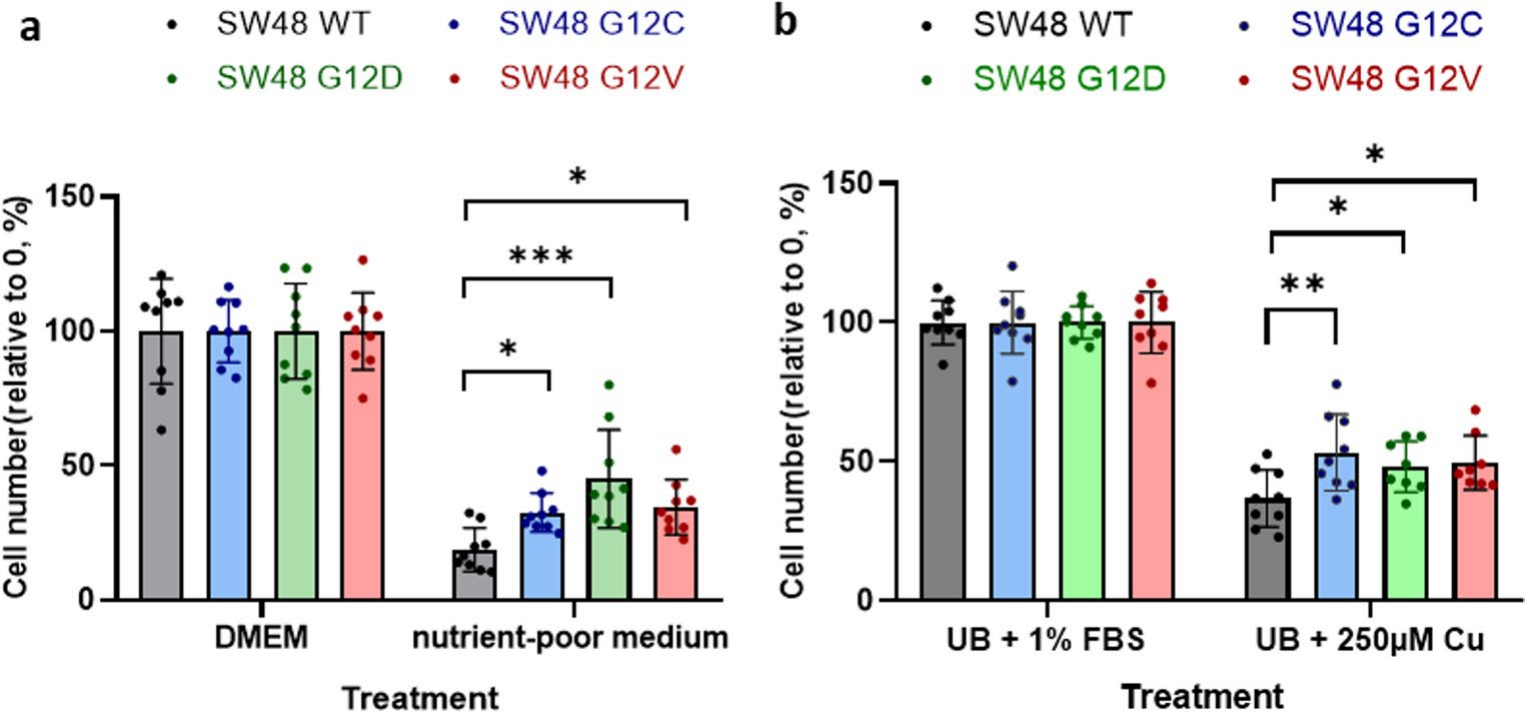
Viability of SW48 cells under nutrient deprivation and acute copper exposure. **a** Viability of KRAS wild-type and mutant SW48 cells after 3 h incubation in nutrient-poor uptake buffer. KRAS wild-type cells exhibited lower survival compared to KRAS-mutant cells. Survival rates are expressed relative to cells maintained in complete medium. Data are derived from nine independent experiments. Significance levels were determined using one-way ANOVA with Tukey’s multiple comparisons test: (*p < 0.05, **p < 0.01, ***p < 0.001). **b** Viability of SW48 cells following a 3 h exposure to a high copper concentration (250 µM CuCl₂) in uptake buffer. KRAS wild-type cells displayed lower viability compared to KRAS-mutant cells. Survival rates are expressed relative to cells maintained in uptake buffer supplemented with 1% FBS. Data are derived from nine independent experiments. Significance levels were determined using one-way ANOVA with Tukey’s multiple comparisons test: (*p < 0.05, **p < 0.01, ***p < 0.001)

It is well established that solid tumors exhibit higher tolerance to harsh microenvironments than non-malignant cells [36]. To model nutrient deprivation, culture medium was replaced with uptake buffer (150 mM NaCl, 2.5 mM MgCl_2_, 25 mM HEPES, pH = 7.4), and cells were incubated for 3 h. Survival rates were 18.8 ± 4.5%, 31.7 ± 6.3%, 45.0 ± 6.5%, 34.6 ± 4.2% in SW48 wild-type, G12C, G12D and G12V cells, respectively (Fig. 8a). These results indicate that KRAS-mutant SW48 cells display significantly enhanced survival under nutrient-deprived conditions compared to the KRAS wild-type cells.

Our copper uptake experiments revealed that KRAS-mutant cells exhibit elevated short-term copper influx accompanied by increased copper efflux over longer incubation periods. Together, these findings suggest that KRAS-mutant cells regulate intracellular copper homeostasis through enhanced copper turnover, characterized by intensified export and import relative to wild-type cells. It was previously found that KRAS-mutant cells possess an increased pool of bioavailable, labile copper with high kinetic accessibility [9]. However, the exact intracellular processes and biomolecules responsible for sustaining this pool remain incompletely understood. Metallothioneins [37], glutathione [10], and specific chaperones [38] have all been implicated in copper storage and maintenance of this balanced copper pool.

Based on these observations, we next examined whether KRAS-mutant cells exhibit increased tolerance against acute copper exposure. Following a 3-hour treatment in uptake buffer containing 250 µM CuCl_2_, cell viabilities were 36.7 ± 10.2%, 54.2 ± 14.3%, 48.1 ± 9.1% and 49.6 ± 9.8% for SW48 WT, G12C, G12D and G12V cells, respectively (Fig. 8b). These results confirm that KRAS-mutant SW48 cells have a greater tolerance to high copper concentrations compared to wild-type cells.

### Response to Copper Depletion Induced by Ammonium Tetrathiomolybdate (TTM)

**Fig. 9:**
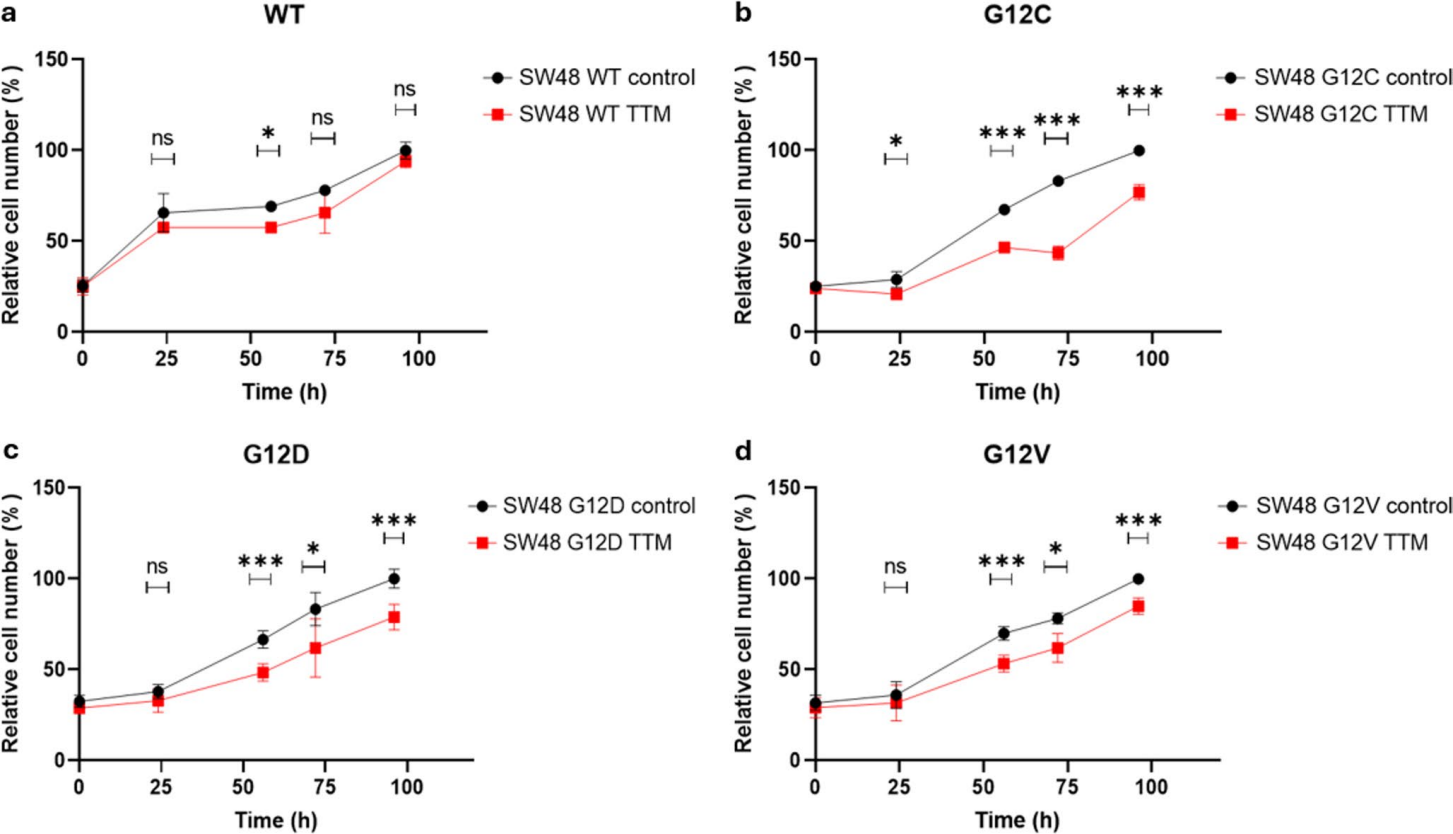
Long-term effects of ammonium tetrathiomolybdate (TTM) on SW48 wild-type and KRAS-mutant cell growth.** a-d** Cell growth of SW48 wild-type and KRAS-mutant cells following treatment with 10 µM TTM. Cell numbers were recorded daily over a 96 h period. TTM treatment resulted in a mild but consistent repression of cell growth in KRAS-mutant cell lines. Significance levels were determined using unpaired two-tailed Student’s *t-tests*: *p < 0.05, **p < 0.01, ***p < 0.001

Based on our findings of altered copper homeostasis in KRAS-mutant cells, we next examined the effect of TTM on SW48 cells to assess potential differences in cell growth and survival. In a 96-hour experiment, cell numbers were measured daily for untreated cells and for cells treated with 10 µM TTM on day 1. Our results show that cell growth was modestly inhibited in KRAS-mutant SW48 cells, irrespective of the specific mutation, compared to wild-type cells. (Fig. 9). As TTM induces copper depletion, this effect may be more pronounced in KRAS-mutant cells, possibly due to their altered pool of bioavailable copper.

### Cytotoxic Effects of Intracellular Copper Chelators on KRAS-Mutant Cells

Cuproplasia describes copper-induced cell growth and proliferation, highlighting the role of elevated copper availability in carcinogenesis. In contrast, excessive intracellular copper accumulation can induce oxidative stress, ultimately triggering cell death in a process termed cuproptosis [39–41]. Aberrant copper homeostasis in tumors may therefore be exploited therapeutically, either by inducing copper-induced cytotoxicity with copper chelators or ionophores, or by targeting cuproplasia via copper depletion strategies [39]. Specifically, copper bioavailability has been identified as a KRAS-specific vulnerability in colorectal cancer [9].

Given the increased copper tolerance observed in KRAS-mutant SW48 cells, we investigated whether these cells also exhibit reduced sensitivity to intracellular copper chelators. The chelators used in this study (neocuproine, DpC, and Dp44mT) are known to exert strong cytotoxic effects in the presence of copper [27, 42, 43]. The corresponding IC_50_ values are summarized in Table 1. KRAS G12C and G12V cells exhibited higher IC_50_ values compared to wild-type and G12D cells, indicating higher tolerance to copper chelators. This suggests that the pool of bioavailable copper levels may be lower in these cells, resulting in reduced formation of cytotoxic copper-chelator complexes. Collectively, these findings support further exploration of copper-targeting strategies in colorectal cancer, with particular consideration of KRAS mutational status.

**Table 1:** Copper chelator sensitivity of SW48 KRAS wild-type and KRAS-mutant cells

	IC50 (µM)		
	neocuproine	DpC	Dp44mT
SW48 WT	1.04 ± 0.09	0.028 ± 0.012	0.023 ± 0.008
SW48 G12C	1.54 ± 0.31**	0.61 ± 0.017*	0.043 ± 0.014*
SW48 G12D	0.91 ± 0.19	0.022 ± 0.005	0.028 ± 0.009
SW48 G12V	1.87 ± 0.43***	0.078 ± 0.01**	0.061 ± 0.025*

IC₅₀ values for neocuproine, DpC, and Dp44mT in SW48 KRAS wild-type and mutant cell lines, determined using the PrestoBlue™ cell viability assay. Data are based on three independent experiments, each performed with three technical replicates. Significance levels were determined using unpaired two-tailed Student’s *t-tests*: *p < 0.05, **p < 0.01, ***p < 0.001 when compared to the KRAS wild-type cells

## Discussion

This study provides insight into the relationship between KRAS mutation status and copper metabolism in SW48 colorectal cancer cells. Although baseline intracellular copper levels differed significantly from wild-type cells in only one case (G12V), pronounced mutation-dependent differences were observed in copper uptake dynamics, transporter expression, and cellular responses to copper manipulation.

Cell proliferation analyses revealed that SW48 G12D cells proliferated significantly more slowly than wild-type, G12C and G12V cell lines. This observation is consistent with a recent comparative study reporting shorter relative doubling times in KRAS G12V compared to G12D cells [44]. Clinical evidence further supports these differences: a retrospective analysis of 495 patients with KRAS-mutant advanced or recurrent colorectal cancer identified G12C and G12V mutations as being associated with the poorest overall survival among KRAS codon 12 variants [45]. In contrast, this study, and an independent review report a more favorable prognosis for KRAS G12D mutations [46]. Together, these findings suggest that distinct KRAS point mutations drive divergent tumor behaviors, likely through mutation-specific intracellular signaling and metabolic pathways.

Copper uptake and intracellular regulation are clinically relevant, as altered copper homeostasis has been implicated in cancer development and progression. Analysis of mRNA levels of copper transporters in colorectal cancer specimens demonstrated a strong association between transporter expression and recurrence-free survival, especially in KRAS-mutant cases. This applied both to CTR1, the primary copper importer, and ATP7A, which mediates copper efflux. While CTR1 is the dominant copper uptake pathway, CTR1-deficient cells retain approximately 30% normal copper uptake capacity [47], indicating the presence of alternative uptake mechanisms. Moreover, copper uptake is influenced not only by CTR1 expression but also by intracellular copper chaperones (ATOX1, CCS) and glutathione levels (GSH) [48], highlighting the complexity of copper trafficking regulation. In KRAS-mutant colorectal cancer, CTR1 expression is generally low, and has been reported to be dispensable for tumor growth and cell fitness [9], consistent with our results showing no significant differences in CTR1 expression among KRAS-mutant subtypes.

In colorectal cancer, increased serum and tissue copper levels are often associated with tumor growth and proliferation [21]. Several clinical studies have reported increased serum copper levels in CRC patients, particularly in advanced disease stages, with strong correlations to TNM stage [22, 23]. A pilot study investigating epithelial-mesenchymal transition (EMT) also found elevated serum copper and reduced serum albumin levels in CRC patients; however, paradoxically, it observed reduced copper levels in tumor tissue together with decreased CTR1 expression [24]. These findings suggest that dysregulated copper trafficking, rather than absolute copper levels, may contribute to tumor progression and metastatic potential. Indeed, reports on copper levels in KRAS-mutant tumors remain conflicting, with studies describing increased tissue copper [25, 26], elevated intracellular copper measured by ICP-MS [12], reduced Cu(I) pools, or no significant change in total copper content [10]. Given this variability and the established importance of copper homeostasis in KRAS-driven colorectal cancer [9], a comprehensive analysis of copper trafficking of our SW48 cell model was warranted.

Our data reveal mutation-specific alterations in intracellular copper handling. Notably, SW48 G12V cells exhibited significantly lower basal copper levels compared with wild-type cells, suggesting enhanced copper export or reduced retention. Under standard culture conditions, copper uptake was negligible across all KRAS-mutant lines; however, short-term copper uptake increased in G12C and G12V cells under nutrient-deprived conditions. This was accompanied by elevated ATP7A expression in these mutants, consistent with a compensatory increase in copper efflux that may account for the reduced intracellular copper levels observed following prolonged exposure. In contrast, G12D cells did not show significant changes in ATP7A expression or copper handling relative to wild-type cells, underscoring mutation-specific differences in copper homeostasis. Importantly, similar trends were observed in colorectal cancer specimens from TCGA, supporting the translational relevance of our in vitro findings.

It has been established, that different KRAS mutations can result in distinct biological and clinical phenotypes [49]. Specific amino acid substitutions (e.g., G12D, G12V, G13D, Q61R) can modify KRAS GTPase activity and its binding preferences for effector molecules, thereby shifting the balance between the RAF–MEK–ERK, PI3K–AKT, and RAL signaling pathways [50]. Co-occurring mutations in genes such as TP53, CDKN2A, SMAD4, and PIK3CA, can also alter signal transduction. The signaling characteristic of G12V cells specifically favors the RalGDS and MAPK/ERK (via Raf-1) signaling pathways, whereas the G12D mutant promotes a more pronounced activation of the PI3K-AKT-mTOR signaling pathway [51]. Based on these findings, it is not unexpected that the greatest differences are observed between the G12D and G12V mutants.

The enhanced survival of KRAS-mutant cells under nutrient deprivation and their increased tolerance to acute copper exposure suggest an adaptive advantage conferred by these mutations. Such adaptations may involve the coordinated regulation of copper transporters, intracellular chaperones, and oxidative stress defense mechanisms [10, 37, 38]. In wild-type KRAS cells, signaling through pathways such as PI3K–mTOR or MAPK is largely dependent on upstream growth factor stimulation provided by components of FBS (e.g., EGF, FGF, VEGF). Consequently, deprivation of FBS leads to attenuation of these signaling cascades, resulting in reduced metabolic activity and survival, features closely connected to the PI3K-mTOR pathway. In contrast, cells harboring oncogenic KRAS mutations (G12C, G12D, G12V) show constitutive activation of KRAS, maintaining downstream signaling independently of extracellular growth factor input [52]. This constitutive activity sustains signaling through key pathways such as PI3K–mTOR, which are critical not only for metabolic regulation but also for the activation of pro-survival signaling programs, even under nutrient- and serum-deprived conditions [50]. In Fig. 8, cells were subjected to short-term (3-hour) deprivation conditions with or without Cu ions. While this timeframe may limit the extent of transcriptional adaptation, it is sufficient to reveal differences in acute metabolic responses. Cell viability was assessed using the PrestoBlue assay, which primarily reflects mitochondrial metabolic activity. Therefore, the higher viability observed in KRAS-mutant cells under nutrient and serum-deprived/reduced conditions likely reflects sustained mitochondrial function, enhanced metabolic activity, and continued activation of survival signaling pathways driven by constitutive KRAS signaling.

In our study of the KRAS-mutant model, we did not observe any further increase in macropinocytosis in any of the mutant cell lines compared to the control cells. This finding contrasts with numerous previous studies reporting increased macropinocytosis in KRAS-mutant cells [35]. This difference may reflect variations in the genetic background of the experimental models. It is known that macropinocytosis can be stimulated not only by KRAS mutations but also by other oncogenic alterations, such as APC mutations, which activate the Wnt signaling pathway [53]. It has been demonstrated that mutations in APC or Axin, as well as activation of the Wnt pathway, can also enhance macropinocytosis activity. As the control cell line (SW48) already carries an APC mutation, macropinocytosis is likely constitutively active and therefore not further inducible by KRAS mutation. In summary, this result highlights the importance of contextual genetic variation in interpreting experimental results. It suggests that KRAS mutation alone does not necessarily elevate macropinocytosis, its effect depends on the cell’s broader oncogenic environment.

Several small biomolecules have been developed to modulate copper metabolism [54], with copper chelators representing a particularly promising therapeutic strategy. Ammonium tetrathiomolybdate (TTM) is a well-characterized copper chelator that has been used clinically for the treatment of Wilson’s disease [55, 56]. Because copper is known to enhance angiogenesis [57], TTM has been widely applied in preclinical and clinical studies to deplete copper in tumor tissues and thereby suppress neovascularization. TTM has been reported to enhance cisplatin sensitivity in breast cancer [58], and to reduce pulmonary metastasis in preclinical models [59]. Moreover, TTM administration significantly reduced tumor growth in mice bearing BRAF-driven mutant tumors [60]. Consistently, studies using 3D spheroid models demonstrated that TTM inhibits cell proliferation in BRAF-mutant, but not in BRAF wild-type, cells [61].

TTM has been shown to significantly suppress the growth of head and neck squamous cell carcinoma in an orthotopic murine model [62] and to inhibit the growth of melanoma cell lines resistant to BRAF or MEK1/2 inhibitors [63]. In SW48 cells TTM modestly inhibited proliferation across all KRAS genotypes, suggesting that its therapeutic potential may be greatest in combination with other targeted approaches in KRAS-mutant tumors.

Finally, the differential sensitivity of KRAS-mutant cells to intracellular copper chelators such as neocuproine and DpC highlights mutation-specific vulnerabilities. The elevated IC_50_ values observed in G12C and G12V cells suggest more efficient regulation of intracellular copper pools, potentially limiting he efficacy of copper chelation as a monotherapy while simultaneously pointing to rational combination strategies.

## Conclusion

This study demonstrates mutation-specific alterations in copper homeostasis in KRAS-mutant SW48 colorectal cancer cells. Among the variants examined, G12C and particularly G12V cells exhibited phenotypes distinct from both wild-type and G12D cells. SW48 G12V cells showed the highest short-term copper uptake, accompanied by the highest expression of the copper transporter ATP7A, while CTR1 and DMT1 levels did not differ significantly among the cell lines. Enhanced copper turnover, characterized by increased influx and efflux, and greater tolerance to both acute copper exposure and intracellular copper chelators, were observed in G12C and G12V cells. Notably, reduced basal copper content was unique to G12V cells and was not observed in G12C mutants.

Collectively, these findings indicate that KRAS mutation subtype influences copper trafficking, intracellular bioavailable copper pools, and sensitivity to copper-targeting interventions. Our results highlight the importance of considering KRAS mutation-specific differences when designing therapeutic strategies aimed at modulating copper homeostasis in colorectal cancer and support the potential of mutation-informed precision approaches for copper-directed therapies.

## Supplementary Information

Below is the link to the electronic supplementary material.

Supplementary Material 1 (DOCX 358 KB)

## Data Availability

The data presented in this article may be provided by the corresponding author on reasonable request.
