# Supplementary material for: Subtype-Specific Alterations in Copper Trafficking Associated with KRAS Mutations in Isogenic Colorectal Cancer Cell Lines
Source: Biol Trace Elem Res. 2026 Jun 2;204(9):7038–53. doi: 10.1007/s12011-026-05171-w (PMC13437747; doi:10.1007/s12011-026-05171-w)
Supplement: Supplementary file 1 [file 12011_2026_5171_MOESM1_ESM.docx]

**Supplementary material**

**Subtype-specific alterations in copper trafficking associated with KRAS mutations in isogenic colorectal cancer cell lines**

**List of Supplemental Figures**

**Figure S1 a** Uncropped image of western blot that correspond to Fig 5a with molecular weight marker **b** Uncropped image of western blot showing the line of β-aktin shown in Fig 5a with molecular weight marker **c** Uncropped image of western blot that correspond to Fig 5b with molecular weight marker **d** Uncropped image of western blot showing the line of β-aktin shown in Fig 5b with molecular weight marker

**Figure S2 a** Uncropped image of western blot that correspond to Fig 6a with molecular weight marker **b** Uncropped image of western blot showing the line of β-aktin shown in Fig 6a with molecular weight marker **c** Uncropped image of western blot that correspond to Fig 6b with molecular weight marker **d** Uncropped image of western blot showing the line of β-aktin shown in Fig 6b with molecular weight marker

**b**

**d**

**c**

**a**

**Supplementary Figure 1**

**d**

**c**

**b**

**a**

**Supplementary Figure 2**
